# Risk factors investigation for different outcomes between unilateral and bilateral chronic rhinosinusitis with nasal polyps patients

**DOI:** 10.1002/clt2.12395

**Published:** 2024-09-25

**Authors:** Jianwei Wang, Yu Zhang, Ying Chen, Xinjun Xu, Yujuan Yang, Jiali Yin, Jing Guo, Pengyi Yu, Zhen Liu, Huifang Liu, Ting Zuo, Hongfei Zhao, Yan Hao, Bei Zhang, Xicheng Song

**Affiliations:** ^1^ Qingdao Medical College Qingdao University Qingdao China; ^2^ Department of Otorhinolaryngology, Head and Neck Surgery Yantai Yuhuangding Hospital Qingdao University Yantai China; ^3^ Shandong Provincial Clinical Research Center for Otorhinolaryngologic Diseases Yantai Shandong China; ^4^ Second Clinical Medicine College Binzhou Medical University Yantai Shandong China; ^5^ Shandong University of Traditional Chinese Medicine Jinan Shandong China; ^6^ Department of Immunology Medical College of Qingdao University Qingdao China

**Keywords:** bilateral, chronic rhinosinusitis with nasal polyps, disease control, recurrence, unilateral

## Abstract

**Background:**

Studies involving chronic rhinosinusitis with nasal polyps (CRSwNP) have mostly focused on bilateral cases, making unilateral CRSwNP inadequately recognized. This study examined the differences in clinical characteristics, outcomes, and risk factors for poor outcomes between unilateral and bilateral CRSwNP to facilitate a better assessment in the two groups.

**Methods:**

Demographic information, tissue and blood cells, endoscopic scores, Lund‐Mackay scores, recurrence rates, and disease control conditions were compared between 310 unilateral and 596 bilateral CRSwNP patients. Furthermore, the stepwise regression multivariate Cox proportional hazard models were performed to generate risk factors for poor outcomes in the two groups.

**Results:**

Bilateral cases exhibited higher rates of smoking, AR, and asthma comorbidities, along with higher numbers of tissue eosinophils and blood inflammatory cells when compared to unilateral patients. Endoscopic nasal polyp score, total computed tomography (CT) score (with scores for each sinus cavity), and adjusted CT scores were significantly higher in the bilateral group, except for a markedly higher adjusted maxillary score in the unilateral group. Furthermore, significantly higher proportions of bilateral patients experienced nasal polyp recurrence, uncontrolled status, and most disease control‐related symptoms at follow‐up. The primary risk factors for poor outcomes were asthma, tissue eosinophils, and total CT score in the bilateral group and blood basophils in the unilateral group.

**Conclusions:**

Bilateral CRSwNP patients experience worse disease severity and outcomes than their unilateral counterparts. Primarily, asthma, tissue eosinophils, and total CT score were risk factors for poor outcomes in bilateral CRSwNP patients, with blood basophils in unilateral cases.

## 1 INTRODUCTION

Chronic rhinosinusitis with nasal polyps (CRSwNP) is an inflammatory outgrowth of sinonasal mucosa that affects 1%–4% of the general adult population.^1^ Although bilateral nasal polyps are more common, unilateral CRSwNP is also frequently encountered. A previous study demonstrated better objective surgical outcomes in unilateral CRSwNP and suggested that the developmental mechanisms of unilateral and bilateral CRSwNP may differ. However, that study had a significant disparity in patient numbers between the two groups,^2^ and there remains a notable lack of research exploring unilateral CRSwNP. Consequently, there is a dearth of understanding regarding the clinical characteristics and treatment outcomes of unilateral CRSwNP, as well as the distinctions between unilateral and bilateral cases.

Functional endoscopic sinus surgery (FESS) is a safe and effective method for treating CRSwNP patients with persistent symptoms following initial medical interventions. Postoperative recurrence is a primary concern in CRSwNP cases. Nonetheless, the European Position Paper on Rhinosinusitis and Nasal Polyps 2020 (EPOS 2020) highlights that there are several other important issues, such as nasal congestion, rhinorrhea/postnasal drip, facial pain/pressure, olfactory dysfunction, sleep disturbance or fatigue, diseased nasal mucosa, and the need for oral rescue medication, that significantly impact a patient's quality of life after surgery.^3^ Understanding the differences in postoperative improvements and risk factors for different outcomes between unilateral and bilateral CRSwNP cases can aid in specific preoperative assessment and postoperative monitoring. Therefore, this study was conducted to elucidate the differences in disease severity, outcomes, and influence factors for outcome‐related items to deepen our understanding of unilateral and bilateral CRSwNP specifically, aiming to provide some guiding information for clinical work.

## 2 MATERIALS AND METHODS

### 2.1 Patients

Data from CRSwNP patients recorded at Yantai Yuhuangding Hospital between January 2016 and June 2021 were collected and analyzed. These patients continued to experience persistent nasal symptoms despite receiving at least 1 month of regular medical treatment. CRSwNP was diagnosed by endoscopic examination according to criteria recommended by the European Position Paper on Rhinosinusitis and Nasal Polyps 2012.^4^ In this study, the unilateral CRSwNP was defined as the endoscopic score of nasal polyps ≥1 in only one nasal cavity, with no hidden nasal polyps, mucosal edema, mucous scar, or mucous ulceration observed in the contralateral nasal cavity under the endoscopic or computed tomography (CT) examination and during operation.^3^ The bilateral CRSwNP was defined as the endoscopic score of nasal polyps ≥1 in each nasal cavity.^3^ All patients included in the study were aged 18 years or older and underwent FESS performed by experienced otolaryngologists with >10 years of practice. Following surgery, the patients received standard treatment consisting of nasal corticosteroid spray and saline irrigation for a period ranging from 3 to 6 months. Individuals with conditions such as non‐steroidal anti‐inflammatory drug (NSAID)‐exacerbated respiratory disease (N‐ERD), fungal sinusitis, odontogenic sinusitis, antrochoanal polyp, benign or malignant tumors, cystic fibrosis, ciliary dysfunction, or immune deficiencies were excluded from the study. Patients who were lost to follow‐up via phone contact were also excluded. Informed consent was obtained from all participants, and the study protocol was approved by the Ethics Committee of Yantai Yuhuangding Hospital at Qingdao University (2024‐219).

### 2.2 Clinical characteristics

Preoperative information was collected, including gender, age, smoking and alcohol habits, comorbid allergic rhinitis (AR) or asthma, and prior nasal surgery. Additionally, data such as results of routine blood examinations, the endoscopic nasal polyps score, and the Lund–Mackay CT score before surgery were gathered. AR was diagnosed based on typical allergic symptoms combined with a positive skin prick test (usually ≥3 mm wheal diameter) or a positive specific serum IgE (usually ≥0.35 kU/L).^5^ Similarly, asthma was diagnosed according to Global Initiative for Asthma recommendations, taking into consideration symptoms such as wheezing, coughing, shortness of breath, or chest tightness, accompanied by positive airway reversibility.^6^ Endoscopic nasal polyps were graded based on their location and extending extent during nasal endoscopic examination, and with the following scoring system: polyps located within the middle meatus (score = 1), polyps extending out the middle meatus (score = 2), large polyps reaching the lower border of the inferior turbinate or medial to the middle turbinate (score = 3), and large polyps blocking the inferior nasal cavity completely (score = 4).^7^ Furthermore, sinus CT scores were assigned using the Lund–Mackay scoring system, with scores indicating no abnormalities (score = 0), partial opacification (score = 1), and total opacification for all sinuses except the ostiomeatal complex (OMC), as well as not occluded (score = 0) and occluded for the OMC (score = 2).^8^

### 2.3 Histopathological evaluation

Samples of nasal polyp tissue obtained during surgery were fixed in 10% formalin and embedded in paraffin. Next, 4‐μm thick sections of tissue specimens were obtained and stained with hematoxylin‐eosin (H&E). Subsequently, two pathologists who were blinded to the clinical data independently counted the numbers of eosinophils, neutrophils, plasma cells, and lymphocytes in five randomly selected high power fields (HPFs, ×400) under a microscope. The ratio of each type of cell to the total number of inflammatory cells was then calculated.

### 2.4 Follow‐up

During the follow‐up session, which was conducted over the phone and ranged from 21 to 86 months (interquartile range: 46.00 [34.50, 51.00] months), patients were asked questions pertaining to the recurrence of nasal polyps and their disease control status as recommended by EPOS 2020 guidelines.^3^ Any patient self‐reported recurrence of nasal polyps was subsequently confirmed by endoscopic examination during postoperative consultations. Patients with unilateral CRSwNP at admission but showed nasal polyp recurrence in both nasal cavities at follow‐up were excluded because of the paradox of classifying them into one specific group according to our grouping method. Disease control was assessed based on the presence or absence of symptoms such as nasal congestion, rhinorrhea/postnasal drip, facial pain/pressure, olfactory dysfunction, sleep disturbance or fatigue, diseased nasal mucosa, and the use of oral rescue medication in the 6 months preceding the follow‐up. The reported diseased nasal mucosa was determined to be present only when confirmed by endoscopic examination. Patients without bothersome symptoms were classified as controlled, those experiencing one or two of the aforementioned symptoms were categorized as partly controlled, and individuals experiencing at least three of these symptoms were deemed uncontrolled.^3^

### 2.5 Statistical analysis

All data were analyzed using IBM SPSS Statistics for Windows, Version 24.0 software (IBM Corp). Continuous variables were assessed for a non‐normal distribution using the Shapiro‐Wilk test, and their values are expressed as a median with interquartile ranges. To compare between‐group differences, the Mann–Whitney *U* test was used for unpaired comparisons. The risk accumulation curves were generated using Prism GraphPad 9.0, and the hazard ratio for bilateral group outcomes compared to unilateral patients was analyzed via log‐rank analysis. To enhance our understanding of sinonasal severity attributable to unilateral and bilateral nasal polyps, all sinus CT scores and nasal polyp scores were adjusted as unilateral. In detail, to make the comparisons between unilateral and bilateral CRSwNP patients more comparable and reasonable. We adjusted CT scores as follows: a specific sinus score = a sum of bilateral scores if the inflammation affected the unilateral sinus, or a specific sinus score = a sum of bilateral scores/2 if the inflammation affected the bilateral sinuses. The same adjustment was used for the endoscopic nasal polyp score: the adjusted score = the total score if only one nasal cavity affected or the adjusted score = the total score/2 if both nasal cavities affected. Dichotomous variables were analyzed using the Chi‐square test or Fisher's exact test for group comparisons. After that, the stepwise regression multivariate Cox proportional hazard models were performed to generate clinical indicators for separately assessing surgical outcomes in unilateral and bilateral groups, with all the demographic information and clinical characteristics being subjected to the analyses. A *p*‐value <0.05 was considered to be statistically significant.

## 3 RESULTS

As shown in Table 1, 310 patients with unilateral CRSwNP and 596 with bilateral CRSwNP were analyzed. The follow‐up periods for the two groups showed no statistically significant difference.

**TABLE 1:** Comparison of clinical characteristics in the unilateral and bilateral CRSwNP groups.

	Unilateral CRSwNP (*n* = 310)	Bilateral CRSwNP (*n* = 596)	*p*‐value
Follow‐up period (mon)	46.50 (38.00, 53.00)	46.00 (33.00, 51.00)	0.123
Gender, male (%)	195 (62.90%)	429 (71.98%)	0.005
Age (years)	51.00 (38.00, 62.00)	51.00 (42.00, 59.00)	0.982
Smoking, *n* (%)	80 (25.81%)	214 (35.91%)	0.002
Alcohol, *n* (%)	76 (24.52%)	165 (27.68%)	0.306
AR, *n* (%)	15 (4.84%)	121 (20.30%)	<0.001
Asthma, *n* (%)	28 (9.03%)	133 (22.32%)	<0.001
Prior surgery, *n* (%)	24 (7.74%)	105 (17.62%)	<0.001
Total tissue inflammatory cell count/HPF	146.00 (101.55, 210.90)	153.45 (114.20, 222.90)	0.130
Tissue eosinophil count/HPF	13.40 (2.25, 29.79)	30.27 (14.95, 66.55)	<0.001
Tissue eosinophil ratio (%)	10.94 (1.77, 20.70)	22.01 (12.35, 40.47)	<0.001
Tissue neutrophil count/HPF	10.60 (2.00, 17.15)	8.63 (1.40, 16.17)	0.149
Tissue neutrophil ratio (%)	7.36 (1.89, 11.15)	5.87 (1.14, 10.63)	0.029
Tissue lymphocyte count/HPF	84.00 (42.25, 119.11)	79.80 (39.15, 105.81)	0.083
Tissue lymphocyte ratio (%)	59.61 (40.98, 69.79)	51.72 (32.14, 65.49)	<0.001
Tissue plasmocyte count/HPF	17.50 (10.00, 40.75)	13.92 (9.13, 37.05)	0.020
Tissue plasmocyte ratio (%)	14.72 (7.38, 29.52)	10.53 (6.42, 21.64)	<0.001
Blood eosinophil count (×10^9^/L)	0.11 (0.06, 0.22)	0.28 (0.16, 0.41)	<0.001
Blood eosinophil ratio (%)	1.90 (1.10, 3.68)	4.30 (2.40, 6.50)	<0.001
Blood basophil count (×10^9^/L)	0.03 (0.02, 0.05)	0.04 (0.03, 0.06)	<0.001
Blood basophil ratio (%)	0.60 (0.40, 0.80)	0.70 (0.50, 0.90)	<0.001
Blood neutrophil count (×10^9^/L)	3.30 (2.58, 4.02)	3.35 (2.63, 4.25)	0.136
Blood neutrophil ratio (%)	55.30 (49.18, 60.40)	53.65 (47.40, 60.40)	0.062
Blood lymphocyte count (×10^9^/L)	1.91 (1.62, 2.32)	2.00 (1.65, 2.47)	0.045
Blood lymphocyte ratio (%)	34.25 (28.93, 38.98)	32.70 (27.60, 38.60)	0.066
Endoscopic score	2.00 (1.00, 3.00)	4.00 (3.00, 5.00)	<0.001
Adjusted endoscopic score	2.00 (1.00, 3.00)	2.00 (1.50, 2.50)	0.662
Total CT score	6.00 (4.00, 8.75)	15.00 (10.00, 21.00)	<0.001
*M* score	2.00 (1.00, 2.00)	2.00 (2.00, 3.00)	<0.001
OMC score	2.00 (0.00, 2.00)	4.00 (4.00, 4.00)	<0.001
Total ethmoid sinus score	2.00 (1.00, 4.00)	9.00 (6.00, 12.00)	<0.001
AE score	2.00 (1.00, 2.00)	4.00 (2.00, 4.00)	<0.001
PE score	1.00 (0.00, 2.00)	2.00 (2.00, 4.00)	<0.001
*S* score	0.00 (0.00, 0.00)	1.00 (0.00, 3.00)	<0.001
*F* score	0.00 (0.00, 1.00)	2.00 (0.00, 4.00)	<0.001
*E*/*M* ratio	1.33 (1.00, 1.67)	2.00 (1.67, 2.50)	<0.001
Adjusted total CT score	5.00 (3.00, 7.00)	7.50 (5.00, 10.50)	<0.001
Adjusted *M* score	1.50 (1.00, 2.00)	1.00 (1.00, 1.50)	<0.001
Adjusted OMC score	1.50 (0.00, 2.00)	2.00 (2.00, 2.00)	<0.001
Adjusted total ethmoid sinus score	2.00 (1.00, 2.50)	4.50 (3.00, 6.00)	<0.001
Adjusted AE score	1.00 (1.00, 2.00)	2.00 (1.00, 2.00)	<0.001
Adjusted PE score	0.50 (0.00, 1.00)	1.00 (1.00, 2.00)	<0.001
Adjusted *S* score	0.00 (0.00, 0.00)	0.50 (0.00, 1.50)	<0.001
Adjusted *F* score	0.00 (0.00, 1.00)	1.00 (0.00, 2.00)	<0.001

Abbreviations: AE score, Anterior ethmoid sinus score; CRSwNP, chronic rhinosinusitis with nasal polyps; CT, computed tomography; *E*/*M* ratio, ethmoid sinus score/maxillary sinus score; *F* score, frontal sinus; HPF, high powered field; *M* score, maxillary sinus score; OMC, ostiomeatal complex; PE score, posterior ethmoid sinus score; *S* score, sphenoid sinus score.

In comparison to the unilateral group, the bilateral group had significantly higher proportions of males, smokers, individuals with AR comorbidity, asthma comorbidity, and those with prior nasal surgery (all *p*‐values <0.05). Additionally, the values for tissue eosinophil count and ratio, blood eosinophil count and ratio, blood basophil count and ratio, and blood lymphocyte count were notably higher in the bilateral group when compared to the unilateral group (all *p*‐values <0.05). Conversely, the unilateral group had significantly higher ratios of tissue neutrophils, tissue lymphocytes, and tissue plasmocytes, as well as a significantly higher numbers of tissue plasmocytes, when compared with the bilateral group (all *p*‐values <0.05) (Table 1).

Additionally, the bilateral CRSwNP patients had significantly higher scores across various symptoms associated with clinical indicators when compared with their unilateral counterparts. Specifically, the bilateral patients had higher endoscopic score, total CT score, and scores for individual sinus regions such as maxillary (*M* score), OMC, total ethmoid, anterior ethmoid (AE score), posterior ethmoid (PE score), sphenoid (*S* score), and frontal sinus (*F* score) (all *p*‐values <0.05). Considering that bilateral disease may have contributed to the overall increased scores in this group, adjusted scores were computed to account for potential confounding factors. The results revealed significantly higher adjusted total CT score, adjusted OMC score, adjusted total ethmoid sinus score, adjusted AE score, adjusted PE score, adjusted *S* score, and adjusted *F* score in the bilateral group when compared to the unilateral group (all *p*‐values <0.05). In contrast, the adjusted *M* score was significantly lower in the bilateral group when compared with the unilateral group (*p* < 0.05), and there was no significant difference in the adjusted endoscopic score between the two groups (Table 1).

**FIGURE 1:**
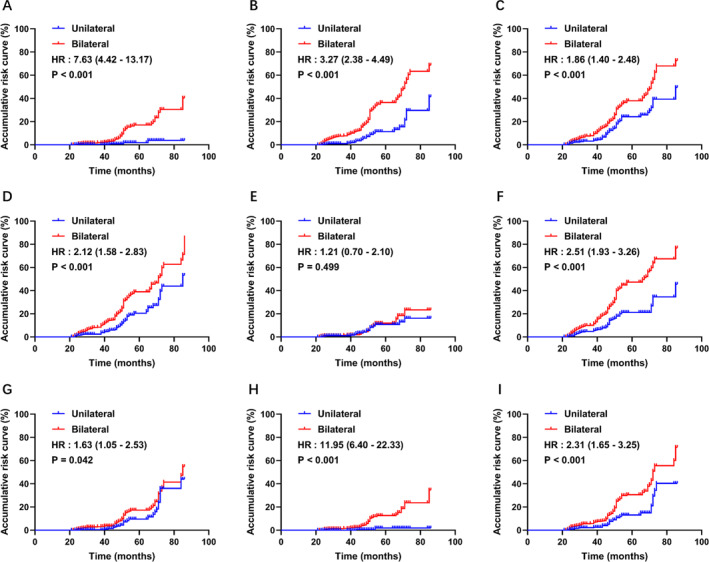
Risk accumulation curves for assessing the hazard ratio for bilateral group outcomes compared with unilateral patients. The outcomes were as follows: (A) Re‐EESS/recurrence, (B) uncontrolled outcome, (C) nasal congestion, (D) rhinorrhea/postnasal drip, (E) facial pain/pressure, (F) olfactory dysfunction, (G) sleep disorder/fatigue, (H) diseased nasal mucosa, and (I) oral rescue medication requirement during the preceding 6 months. EESS, extended endoscopic sinus surgery; HR, hazard ratio.

In terms of surgical outcomes, our study revealed considerably higher rates of nasal polyp recurrence and uncontrolled disease in the bilateral group (all *p*‐values <0.05). Additionally, a notably greater proportion of patients in the bilateral group reported experiencing nasal congestion, rhinorrhea/postnasal drip, olfactory dysfunction, diseased nasal mucosa, or the need for oral rescue medication within the previous 6 months (all *p*‐values <0.05). However, there was no statistical difference between the groups in terms of the proportions of patients experiencing facial pain/pressure, sleep disorders, or fatigue, although a higher trend was shown in the bilateral group, as illustrated in Table 2. All the patients who suffered from nasal polyp recurrence accepted a second surgery (4 in the unilateral group and 51 in the bilateral). The risk accumulation curves for all the surgical outcomes were generated to show risk for outcomes between groups, and it was revealed that the risks were significantly higher in the bilateral group for nearly all outcomes except the facial pain/pressure (Figure 1).

**TABLE 2:** Comparison of outcomes in the unilateral and bilateral CRSwNP groups.

Outcomes	Unilateral CRSwNP (*n* = 310)	Bilateral CRSwNP (*n* = 596)	*p*‐value
Recurrence, *n* (%)	4 (1.29%)	51 (8.56%)	<0.001
Disease control			<0.001
Controlled, *n* (%)	208 (67.10%)	297 (49.83%)	
Partly controlled, *n* (%)	77 (24.84%)	160 (26.85%)	
Uncontrolled, *n* (%)	25 (8.06%)	139 (23.32%)	
Nasal congestion, *n* (%)	48 (15.48%)	153 (25.67%)	<0.001
Rhinorrhea/postnasal drip, *n* (%)	42 (13.55%)	152 (25.50%)	<0.001
Facial pain/pressure, *n* (%)	18 (5.81%)	37 (6.21%)	0.810
Olfactory dysfunction, *n* (%)	45 (14.52%)	196 (32.89%)	<0.001
Sleep disorder/fatigue, *n* (%)	23 (7.42%)	61 (10.23%)	0.166
Diseased nasal mucosa, *n* (%)	2 (0.65%)	40 (6.71%)	<0.001
Oral rescue medication requirement during the preceding 6 months, *n* (%)	29 (9.35%)	114 (19.13%)	<0.001

Abbreviation: CRSwNP, chronic rhinosinusitis with nasal polyps.

Since the clinical symptom severity and disease outcomes revealed remarked differences between patients with unilateral and bilateral CRSwNP, we further operated backward stepwise multivariate Cox proportional hazard models in order to identify some practical clinical indicators for assessing their outcomes in advance separately.

For the primary outcomes of nasal polyp recurrence and uncontrolled status, elder age was identified as a protective factor, with asthma and higher total CT score being identified as risk factors for bilateral CRSwNP patients. Furthermore, total tissue inflammatory cell count, and tissue eosinophil and lymphocyte ratios were shown to be risk factors, along with blood lymphocyte ratio being a protective factor for uncontrolled disease condition in bilateral CRSwNP patients. In unilateral CRSwNP patients, the risk factors were found to be blood basophil and neutrophil ratios, blood lymphocyte count, and *E*/*M* ratio, with the prospective factor being endoscopic score for uncontrolled status. However, no significant factor for nasal polyp recurrence was found in the unilateral group (Table 3).

**TABLE 3:** Influence factors on primary outcomes in the unilateral and bilateral CRSwNP groups.

Primary outcomes	Influence factors	Unilateral CRSwNP			Bilateral CRSwNP		
		HR	95% CI	*p*‐value	HR	95% CI	*p*‐value
Recurrence	Age	‐	‐	‐	0.956	0.934–0.978	<0.001
	Asthma	‐	‐	‐	2.128	1.155–3.918	0.015
	Total CT score	‐	‐	‐	1.056	1.004–1.109	0.033
Uncontrolled	Age	‐	‐	‐	0.972	0.959–0.986	<0.001
	Asthma	‐	‐	‐	1.513	1.029–2.224	0.035
	Total tissue inflammatory cell count/HPF	‐	‐	‐	1.001	1.000–1.001	0.007
	Tissue eosinophil ratio (%)	‐	‐	‐	1.016	1.004–1.029	0.010
	Tissue lymphocyte ratio (%)	‐	‐	‐	1.015	1.003–1.027	0.017
	Blood lymphocyte ratio (%)	‐	‐	‐	0.976	0.956–0.996	0.022
	Blood basophil ratio (%)	1.587	1.200–2.099	0.001	‐	‐	‐
	Blood neutrophil ratio (%)	1.095	1.017–1.178	0.016	‐	‐	‐
	Blood lymphocyte count (×10^9^/L)	2.661	1.234–5.740	0.013	‐	‐	‐
	E/*M* ratio	1.508	1.062–2.140	0.022	‐	‐	‐
	Endoscopic score	0.522	0.310–0.879	0.014	‐	‐	‐
	Total CT score	‐	‐	‐	1.052	1.021–1.085	0.001

Abbreviations: CI, confidence interval; CRSwNP, chronic rhinosinusitis with nasal polyps; *E*/*M* ratio, ethmoid sinus score/maxillary sinus score.

As the disease control status was assessed based on seven different items at follow‐up, we further explored the significant influence factors for each item.

In the unilateral group, the risk factors were identified to be blood basophil count and ratio for nasal congestion; alcohol, total tissue inflammatory cell count and blood basophil ratio for rhinorrhea/postnasal drip; alcohol, blood basophil and neutrophil ratios, blood lymphocyte count for facial pain/pressure; blood basophil ratio for olfactory dysfunction; tissue eosinophil and neutrophil counts, blood neutrophil ratio and blood lymphocyte count for sleep disturbance or fatigue; tissue eosinophil ratio, blood basophil ratio, blood neutrophil ratio, *E*/*M* ratio for oral rescue requirement. The protective factors were identified to be age and blood eosinophil ratio for nasal congestion, tissue lymphocyte count, blood eosinophil and lymphocyte ratios, and the endoscopic score for rhinorrhea/postnasal drip, total tissue inflammatory cell count, tissue eosinophil ratio, tissue neutrophil and lymphocyte ratios, endoscopic score for sleep disturbance or fatigue, and blood eosinophil ratio for oral rescue requirement (Table 4).

**TABLE 4:** Influence factors for disease control‐related outcome items in the unilateral and bilateral CRSwNP groups.

Disease control‐related outcome items	Influence factors	Unilateral CRSwNP			Bilateral CRSwNP		
		HR	95% CI	*p*‐value	HR	95% CI	*p*‐value
Nasal congestion	Age	0.978	0.958–0.999	0.044	0.975	0.963–0.988	<0.001
	Prior surgery, *n* (%)				1.604	1.088–2.363	0.017
	Blood eosinophil ratio (%)	0.809	0.689–0.949	0.009	‐	‐	‐
	Blood basophil count (×10^9^/L)	438767.310	1.762–109259822400.000	0.040	‐	‐	‐
	Blood basophil ratio (%)	1.334	1.018–1.748	0.037	‐	‐	‐
	Blood lymphocyte ratio (%)	‐	‐	‐	0.975	0.956–0.994	0.011
Rhinorrhea/postnasal drip	Age	‐	‐	‐	0.977	0.965–0.990	0.001
	Asthma	‐	‐	‐	1.624	1.128–2.337	0.009
	Alcohol	4.067	1.718–9.629	0.001	‐	‐	‐
	Total tissue inflammatory cell count/HPF	1.003	1.002–1.005	0.001	1.001	1.000–1.001	0.006
	Tissue eosinophil ratio (%)	‐	‐	‐	1.012	1.001–1.024	0.039
	Tissue lymphocyte count/HPF	0.992	0.986–0.997	0.003	‐	‐	‐
	Tissue lymphocyte ratio (%)	‐	‐	‐	1.012	1.000–1.023	0.044
	Blood eosinophil ratio (%)	0.824	0.696–0.976	0.025	‐	‐	‐
	Blood basophil count (×10^9^/L)	‐	‐	‐	8.208	1.074–62.710	0.042
	Blood basophil ratio (%)	1.469	1.125–1.919	0.005	‐	‐	‐
	Blood lymphocyte ratio (%)	0.953	0.913–0.995	0.029	0.975	0.955–0.994	0.011
	Endoscopic score	0.661	0.448–0.974	0.036	‐	‐	‐
	Total CT score	‐	‐	‐	1.030	1.002–1.059	0.038
Facial pain/pressure	Age	‐	‐	‐	0.966	0.940–0.991	0.010
	Alcohol	5.456	1.192–24.970	0.029	‐	‐	‐
	Blood basophil ratio (%)	1.784	1.196–2.661	0.005	‐	‐	‐
	Blood neutrophil count (×10^9^/L)	‐	‐	‐	1.333	1.079–1.648	0.008
	Blood neutrophil ratio (%)	1.084	1.005–1.169	0.036	0.952	0.917–0.989	0.012
	Blood lymphocyte count (×10^9^/L)	2.553	1.248–5.221	0.010			
Olfactory dysfunction	Age	‐	‐	‐	0.981	0.970–0.992	0.001
	Tissue eosinophil ratio (%)	‐	‐	‐	1.012	1.001–1.022	0.026
	Tissue lymphocyte ratio (%)	‐	‐	‐	1.013	1.003–1.023	0.014
	Blood basophil ratio (%)	1.309	1.066–1.608	0.010	‐	‐	‐
	Total CT score	‐	‐	‐	1.053	1.026–1.080	<0.001
Sleep disorder/fatigue	Age	‐	‐	‐	0.970	0.951–0.990	0.003
	Asthma	‐	‐	‐	1.845	1.008–3.377	0.047
	Total tissue inflammatory cell count/HPF	0.991	0.984–0.999	0.024	1.004	1.001–1.007	0.008
	Tissue eosinophil count/HPF	1.014	1.003–1.026	0.012	‐	‐	‐
	Tissue eosinophil ratio (%)	0.944	0.907–0.984	0.006	‐	‐	‐
	Tissue neutrophil count/HPF	1.049	1.006–1.093	0.024	0.968	0.942–0.994	0.017
	Tissue neutrophil ratio (%)	0.873	0.780–0.976	0.017	1.068	1.010–1.130	0.022
	Tissue lymphocyte ratio (%)	0.957	0.927–0.988	0.007	1.017	1.001–1.033	0.041
	Blood neutrophil ratio (%)	1.168	1.075–1.270	<0.001	‐	‐	‐
	Blood basophil count (×10^9^/L)	‐	‐	‐	63.903	6.050–674.931	0.001
	Blood lymphocyte count (×10^9^/L)	3.968	1.661–9.476	0.002	‐	‐	‐
	Endoscopic score	0.391	0.191–0.798	0.010	‐	‐	‐
	Total CT score	‐	‐	‐	1.049	1.002–1.099	0.041
Diseased nasal mucosa	Age	‐	‐	‐	0.959	0.935–0.984	0.001
	Total CT score	‐	‐	‐	1.103	1.038–1.172	0.002
Oral rescue medication requirement	Age	‐	‐	‐	0.970	0.955–0.985	<0.001
	Alcohol	‐	‐	‐	0.543	0.316–0.933	0.027
	Prior surgery	‐	‐	‐	1.677	1.058–2.658	0.028
	Asthma	‐	‐	‐	2.479	1.650–3.724	<0.001
	Tissue eosinophil ratio (%)	1.042	1.007–1.078	0.019	1.023	1.008–1.037	0.002
	Tissue lymphocyte ratio (%)	‐	‐	‐	1.016	1.001–1.030	0.034
	Blood eosinophil ratio (%)	0.765	0.606–0.966	0.025	‐	‐	‐
	Blood basophil ratio (%)	1.642	1.135–2.377	0.009	‐	‐	‐
	Blood neutrophil ratio (%)	1.062	1.009–1.118	0.020	‐	‐	‐
	E/*M* ratio	1.556	1.122–2.157	0.008	0.814	0.668–0.992	0.041
	Total CT score	‐	‐	‐	1.081	1.034–1.129	0.001
	Endoscopic score	‐	‐	‐	0.826	0.699–0.976	0.024

Abbreviations: CI, confidence interval; CRSwNP, chronic rhinosinusitis with nasal polyps; *E*/*M* ratio, ethmoid sinus score/maxillary sinus score.

In the bilateral group, the risk factors were identified to be prior surgery for nasal congestion; asthma, total tissue inflammatory cell count, tissue eosinophil and lymphocyte ratios, blood basophil count, total CT score for rhinorrhea/postnasal drip; blood neutrophil count for facial pain/pressure; tissue eosinophil and lymphocyte ratio, total CT score for olfactory dysfunction; asthma, total tissue inflammatory cell count, tissue neutrophil and lymphocyte ratio, blood basophil count and total CT score for sleep disturbance or fatigue; total CT score for diseased nasal mucosa; prior surgery, asthma, tissue eosinophil and lymphocyte ratios, and total CT score for oral rescue requirement. The protective factors were identified to be age and blood lymphocyte ratio for nasal congestion; age and blood lymphocyte ratio for rhinorrhea/postnasal drip; age and blood neutrophil ratio for facial pain/pressure; age for olfactory dysfunction; age and tissue neutrophil count for sleep disturbance or fatigue; age for diseased nasal mucosa; alcohol, *E*/*M* ratio and endoscopic score for oral rescue requirement (Table 4).

## 4 DISCUSSION

Unilateral CRSwNP has been a less studied aspect of CRSwNP. Previous research has primarily focused on bilateral nasal polyps, with severe CRSwNP typically referring to cases involving bilateral polyps.^9^, ^10^, ^11^ A previous study addressed unilateral nasal polyps and suggested potentially better outcomes than bilateral cases, indicating a distinct progression in the unilateral CRSwNP. However, that study, including only 23 patients with unilateral CRSwNP compared to a larger group with bilateral polyps, lacked convincing findings.^2^ Despite the rarity of unilateral CRSwNP in research studies, such cases are repeatedly encountered in clinical practice. This underscores the necessity to explore the clinical characteristics and postoperative outcomes of unilateral CRSwNP and clarify differences between unilateral and bilateral groups to facilitate a better assessment in advance and more targeted treatment strategies.

In our study, the numbers of patients in the unilateral and bilateral groups were comparable and there was no statistically significant difference in the follow‐up periods between the two groups. Therefore, a more detailed analysis could be conducted, leading to more convincing conclusions.

Our study, in line with previous research findings, supports the common understanding that severe CRSwNP primarily manifests as bilateral, with elevated eosinophils, and is often accompanied by asthma and AR comorbidities as well as a smoking habit.^10^, ^12^, ^13^, ^14^ At the same time, the numbers of various blood inflammatory cell types were significantly higher in the bilateral CRSwNP group, suggesting a more severe symptom burden. Despite a higher total tissue inflammatory cell count, the remarkable eosinophil infiltration observed in the bilateral group (>2‐fold greater than in the unilateral group) may account for the relatively lower numbers and ratios of other inflammatory cell types in the bilateral cases. The endoscopic nasal polyp score and total CT score, along with each type of sinus score, further supported the notion that patients with bilateral CRSwNP experience more severe symptoms. Specifically, adjusted CT scores indicated more severe sinus involvement in nearly all sinuses among the bilateral CRSwNP cases, suggesting potential advanced nasal polyp progression compared with unilateral cases. Notably, our findings showed a higher adjusted *M* score in the unilateral group, contrary to other sinuses types. As it was a previously proposed that an *E*/*M* ratio >2.59 effectively predicts eosinophilic CRSwNP, a relatively higher adjusted *M* score might suggest a lesser severity of nasal polyps.^15^ This discrepancy suggests potential differences in the developmental mechanism and disease progression between the two groups.

A previous study also supported a potential difference in the progression between groups and revealed more favorable objective surgical outcomes in cases of unilateral CRSwNP.^2^ However, another study of chronic rhinosinusitis without nasal polyps demonstrated similar surgical outcomes for unilateral and bilateral cases.^16^ These studies, albeit informative, had limitations as they were conducted with small patient groups affected by unilateral disease, and this may have contributed to inconclusive findings. In contrast, our study represents a significant advancement, as we conducted a comprehensive analysis of a sizable cohort of patients with unilateral CRSwNP, and found notably superior surgical outcomes when compared to their bilateral counterparts. Specifically, the recurrence rate was approximately 7‐fold higher and the uncontrolled condition was nearly 3‐fold higher in the bilateral group. Furthermore, we meticulously evaluated various disease control‐related outcomes, and found that patients in the unilateral group achieved favorable results across all parameters, despite not displaying statistically significant differences in facial pain/pressure, sleep disorder, or fatigue. These detailed analyses provide valuable insights into both the overall progression and specific outcome conditions of the disease in the two groups.

In light of the observed significantly worsened symptoms and surgical outcomes in bilateral cases compared with their unilateral counterparts, we reasonably hypothesized that the assessment and prediction of surgical outcomes for the two patient populations should be performed separately. According to the Cox analyses, asthma comorbidity, tissue eosinophil infiltration, and increased total CT score were the primary risk factors for most outcome items in patients with bilateral CRSwNP. It is consistent with the prior recognition that a higher CT score and asthma comorbidity can lead to poor outcomes, including nasal polyp recurrence and uncontrolled status, in CRSwNP.^17^, ^18^ Studies have also marked tissue eosinophil as an essential indicator and predictor of poor outcomes of CRSwNP, supporting our findings.^19^, ^20^ However, the influence factors identified in the unilateral group were dramatically different. Blood basophils showed a strong association with poor outcomes. Previous studies have reported that blood basophil acts as a risk factor for nasal polyp recurrence and that basophils may be involved in the exacerbated progression of CRSwNP.^21^, ^22^ This reminded us of the importance of further investigation of basophils to reveal the potential mechanisms for triggering nasal polyp formation and exacerbation. Nevertheless, other factors in the unilateral group variated greatly; even the tissue and blood eosinophils seemed to play a less critical role in this group. Although blood neutrophils and lymphocytes were identified as risk factors for some poor outcomes in unilateral CRSwNP, previous studies indicated that their roles in assessing the severity and outcomes in CRSwNP remained unconvincing.^23^, ^24^, ^25^

There are some limitations in this study. First, patients determined by unilateral CRSwNP but showed bilateral polyp recurrence were excluded from this study. A deeper investigation into this patient group can definitely deepen our understanding of the progression mechanism of CRSwNP and associated factors. However, we believe such an exploration should be based on a basis recognition of the difference between unilateral and bilateral CRSwNP since some unilateral CRSwNP may only reoccur in the homolateral nasal cavity with no inflammatory signs in the contralateral nasal cavity throughout the period. Second, it is a single‐centered study, and our findings need to be confirmed in future research.

## 5 CONCLUSION

In comparison to their unilateral counterparts, patients with bilateral CRSwNP experience greater disease severity and worse outcomes. Furthermore, the risk factors were dramatically different between the two groups. Asthma comorbidity, tissue eosinophil infiltration, and increased total CT score were the primary risk factors for poor outcomes in patients with bilateral CRSwNP, with blood basophil being the major risk factor in unilateral CRSwNP patients.

## AUTHOR CONTRIBUTIONS

*Study idea, design and data analysis*: Jianwei Wang and Yu Zhang. *Clinical data screening and collection*: Ying Chen, Xinjun Xu, Yujuan Yang, Jiali Yin, Jing Guo, and Pengyi Yu. *Sample screening and collection*: Zhen Liu and Huifang Liu. *Experiment*: Ting Zuo, Hongfei Zhao, and Yan Hao. *Draft manuscript*: Jianwei Wang, Yu Zhang, Ying Chen, and Xinjun Xu. *Critical review and revision of the manuscript*: Bei Zhang and Xicheng Song. *Final approval*: all authors.

## CONFLICT OF INTEREST STATEMENT

The authors declare no conflicts of interest.

## Data Availability

The data that support the findings of this study are available from the corresponding author upon reasonable request.

## References

[1] Jin Z , Yan B , Zhang L , Wang C . Current and emerging biological therapies for chronic rhinosinusitis with nasal polyps with type 2 inflammation. Expet Opin Invest Drugs. 2023;32(10):909‐919. 10.1080/13543784.2023.2273502 37855222

[2] Lee JY , Byun JY , Shim SS , Lee SW . Outcomes after endoscopic sinus surgery for unilateral versus bilateral chronic rhinosinusitis with nasal polyposis. Am J Rhinol Allergy. 2010;24(3):83‐86. 10.2500/ajra.2010.24.3482 20537280

[3] Fokkens WJ , Lund VJ , Hopkins C , et al. European position paper on rhinosinusitis and nasal polyps 2020. Rhinology. 2020;58(Suppl S29):1‐464. 10.4193/rhin20.600 32077450

[4] Fokkens WJ , Lund VJ , Mullol J , et al. European position paper on rhinosinusitis and nasal polyps 2012. Rhinol Suppl. 2012;23(3):1‐298. 22764607

[5] Wise SK , Lin SY , Toskala E , et al. International consensus statement on allergy and rhinology: allergic rhinitis. Int Forum Allergy Rhinol. 2018;8(2):108‐352. 10.1002/alr.22073 29438600

[6] Global Initiative for Asthma . Global strategy for asthma management and prevention. 2021. www.ginasthma.org

[7] Gevaert P , Calus L , Van Zele T , et al. Omalizumab is effective in allergic and nonallergic patients with nasal polyps and asthma. J Allergy Clin Immunol. 2013;131(1):110‐116.e1. 10.1016/j.jaci.2012.07.047 23021878

[8] Lund VJ , Kennedy DW . Staging for rhinosinusitis. Otolaryngol Head Neck Surg. 1997;117(3 Pt 2):S35‐S40. 10.1016/s0194-5998(97)70005-6 9334786

[9] Dharmarajan H , Falade O , Lee SE , Wang EW . Outcomes of dupilumab treatment versus endoscopic sinus surgery for chronic rhinosinusitis with nasal polyps. Int Forum Allergy Rhinol. 2022;12(8):986‐995. 10.1002/alr.22951 34919344

[10] Bachert C , Han JK , Desrosiers M , et al. Efficacy and safety of dupilumab in patients with severe chronic rhinosinusitis with nasal polyps (LIBERTY NP SINUS‐24 and LIBERTY NP SINUS‐52): results from two multicentre, randomised, double‐blind, placebo‐controlled, parallel‐group phase 3 trials. Lancet. 2019;394(10209):1638‐1650. 10.1016/s0140-6736(19)31881-1 31543428

[11] Fokkens WJ , De Corso E , Backer V , et al. EPOS2020/EUFOREA expert opinion on defining disease states and therapeutic goals in CRSwNP. Rhinology. 2024;0(0):0. 10.4193/rhin23.415 38217529

[12] Zhang Y , Yan B , Shen S , et al. Efficacy and safety of CM310 in severe eosinophilic chronic rhinosinusitis with nasal polyps (CROWNS‐1): a multicentre, randomised, double‐blind, placebo‐controlled phase 2 clinical trial. EClinicalMedicine. 2023;61:102076. 10.1016/j.eclinm.2023.102076 37483544 PMC10359732

[13] Bachert C , Maurer M , Palomares O , Busse WW . What is the contribution of IgE to nasal polyposis? J Allergy Clin Immunol. 2021;147(6):1997‐2008. 10.1016/j.jaci.2021.03.016 33757720

[14] Hutson K , Clark A , Hopkins C , et al. Evaluation of smoking as a modifying factor in chronic rhinosinusitis. JAMA Otolaryngol Head Neck Surg. 2021;147(2):159‐165. 10.1001/jamaoto.2020.4354 33300989 PMC7729579

[15] Meng Y , Lou H , Wang C , Zhang L . Predictive significance of computed tomography in eosinophilic chronic rhinosinusitis with nasal polyps. Int Forum Allergy Rhinol. 2016;6(8):812‐819. 10.1002/alr.21749 27060677

[16] Beswick DM , Mace JC , Chowdhury NI , et al. Comparison of surgical outcomes between patients with unilateral and bilateral chronic rhinosinusitis. Int Forum Allergy Rhinol. 2017;7(12):1162‐1169. 10.1002/alr.22020 28941136 PMC5716933

[17] Vlaminck S , Acke F , Prokopakis E , et al. Surgery in nasal polyp patients: outcome after a minimum observation of 10 years. Am J Rhinol Allergy. 2021;35(4):449‐457. 10.1177/1945892420961964 33019818

[18] Tao X , Chen F , Sun Y , et al. Prediction models for postoperative uncontrolled chronic rhinosinusitis in daily practice. Laryngoscope. 2018;128(12):2673‐2680. 10.1002/lary.27267 30295929

[19] Lou H , Meng Y , Piao Y , Wang C , Zhang L , Bachert C . Predictive significance of tissue eosinophilia for nasal polyp recurrence in the Chinese population. Am J Rhinol Allergy. 2015;29(5):350‐356. 10.2500/ajra.2015.29.4231 26219765

[20] Lou H , Meng Y , Piao Y , et al. Cellular phenotyping of chronic rhinosinusitis with nasal polyps. Rhinology. 2016;54(2):150‐159. 10.4193/rhin15.271 26747641

[21] Brescia G , Barion U , Zanotti C , Giacomelli L , Martini A , Marioni G . The prognostic role of serum eosinophil and basophil levels in sinonasal polyposis. Int Forum Allergy Rhinol. 2017;7(3):261‐267. 10.1002/alr.21885 27992119

[22] Stevens WW , Staudacher AG , Hulse KE , et al. Studies of the role of basophils in aspirin‐exacerbated respiratory disease pathogenesis. J Allergy Clin Immunol. 2021;148(2):439‐449.e5. 10.1016/j.jaci.2021.02.045 33819512 PMC8355049

[23] Kara A , Guven M , Yilmaz MS , Demir D , Elden H . Are neutrophil, platelet and eosinophil‐to‐lymphocyte ratio and red blood cell distribution width can be used for nasal polyposis? Eur Arch Oto‐Rhino‐Laryngol. 2018;275(2):409‐413. 10.1007/s00405-017-4821-3 29192331

[24] Brescia G , Pedruzzi B , Barion U , et al. Are neutrophil‐eosinophil‐and basophil‐to‐lymphocyte ratios useful markers for pinpointing patients at higher risk of recurrent sinonasal polyps? Am J Otolaryngol. 2016;37(4):339‐345. 10.1016/j.amjoto.2016.02.002 27045767

[25] Brescia G , Barion U , Zanotti C , Parrino D , Marioni G . Pre‐ and postoperative blood neutrophil‐to‐lymphocyte and eosinophil‐to‐lymphocyte ratios in patients with sinonasal polyps: a preliminary investigation. Allergy Asthma Proc. 2017;38(5):64‐69. 10.2500/aap.2017.38.4068 28814353

